# Hallucinogen use in the United States, 2021–2023: Diverging trends and subgroup patterns

**DOI:** 10.1016/j.dadr.2026.100410

**Published:** 2026-01-19

**Authors:** Jing-Jer Chen, Carla J. Berg, Y. Tony Yang

**Affiliations:** a Department of Health Policy and Management, Milken Institute School of Public Health, George Washington University, Washington, DC, USA; b Department of Prevention and Community Health, Milken Institute School of Public Health, George Washington University, Washington, DC, USA; c George Washington Cancer Center, George Washington University, Washington, DC, USA; d Center for Health Policy and Media Engagement, School of Nursing, George Washington University, Washington, DC, USA

**Keywords:** Hallucinogens, LSD, Ketamine, Psychedelics, Epidemiology, Substance use trends

## Abstract

**Background:**

While interest in the therapeutic and recreational use of hallucinogens has increased, national surveillance often reports use in aggregate, potentially masking shifting trends among pharmacologically distinct substances. This study assessed trends in specific hallucinogens from 2021 to 2023 and identified correlates of use, with particular attention to subgroup patterns in populations commonly prioritized for prevention and access-focused interventions.

**Methods:**

Using nationally representative NSDUH data (2021–2023; ages ≥12), we estimated annual past-year prevalence of LSD, PCP, ecstasy (MDMA), ketamine, Salvia divinorum, and tryptamines (including DMT). We fit survey-weighted logistic regression models with year (continuous) to assess trends and pooled multivariable models to examine demographic correlates.

**Results:**

Although overall past-year hallucinogen use was stable (2.83 % [95 % CI: 2.52–3.14] in 2021; 2.82 % [2.52–3.12] in 2023), substance-specific trends diverged. LSD declined (aOR per year=0.83, 95 % CI: 0.75–0.93), from 0.88 % (0.72–1.04) in 2021–0.58 % (0.47–0.68) in 2023. Ketamine increased (aOR=1.11, 95 % CI: 1.02–1.21), from 1.61 % (1.42–1.80) to 1.91 % (1.67–2.16). Ecstasy/MDMA and tryptamines were stable, and PCP and Salvia remained rare. Use concentrated among young adults and males; adjusted models indicated higher odds among uninsured respondents and those below the federal poverty level.

**Conclusions:**

Despite stable overall hallucinogen prevalence, significant increases were observed for ketamine alongside declines for LSD, suggesting a shifting landscape of hallucinogen use. Substance-specific monitoring may better inform screening, prevention, and harm-reduction efforts than aggregate hallucinogen indicators, especially as ketamine’s medical availability expands and disparities in access to mental health treatment persist.

## Highlights

• LSD use declined annually 2021–2023 in U.S. NSDUH data.
• Ketamine use rose, notably among males and uninsured adults.
• Divergent trends suggest substance-specific policy responses.
• Education remains strongly associated with hallucinogen use.
• Findings inform targeted harm-reduction and surveillance.

## 1 Introduction

Interest in the therapeutic applications of hallucinogens has grown substantially over the past decade, accompanied by rapid shifts in the regulatory landscape. The FDA approval of esketamine nasal spray for treatment-refractory depression in 2019 marked a watershed moment, representing the first new mechanism of action for depression treatment in decades (Kim et al., 2019). Subsequently, specialized ketamine clinics have proliferated across the United States, offering off-label intravenous infusions for depression, chronic pain, and other conditions (Bloomfield et al., 2023, Mangiacarne, 2025). Concurrently, a wave of psychedelic policy reform has swept across states and municipalities: Denver decriminalized psilocybin in 2019, Oregon legalized psilocybin for therapeutic use in 2020, and Colorado approved similar measures in 2022 (Marks, 2021). These developments have coincided with renewed scientific interest in psychedelic-assisted psychotherapy for treatment-refractory depression, post-traumatic stress disorder, and substance use disorders (Carhart-Harris and Goodwin, 2017).

While hallucinogen use has been rising in recent years, these substances are pharmacologically distinct and may follow divergent trajectories. Classic serotonergic psychedelics like lysergic acid diethylamide (LSD) and psilocybin act as 5-HT2A receptor agonists, producing perceptual alterations lasting 6–12 h (Nichols, 2016). Dissociative agents like ketamine function as N-methyl-D-aspartate (NMDA) receptor antagonists, inducing shorter experiences characterized by detachment (Zanos et al., 2018). Ketamine’s Schedule III status and medical legitimacy contrast sharply with the continued Schedule I classification of classic psychedelics. This heterogeneity extends to less commonly used substances such as phencyclidine (PCP) and Salvia divinorum, which carry substantial acute risks and remain important for public health surveillance (Livne et al., 2022, Walsh et al., 2022), as well as tryptamine psychedelics (e.g., N,N-dimethyltryptamine [DMT]) that produce intense but rapid-onset effects (Walsh et al., 2022).

National trend analyses have documented increasing use of several hallucinogen subtypes among U.S. adults since 2002, including LSD, psilocybin, tryptamines, and ketamine (Yockey et al., 2020, Livne et al., 2022, Walsh et al., 2022, Yang et al., 2025). However, recent preliminary data suggest these divergent regulatory and accessibility factors may be producing shifting patterns within the hallucinogen class: young adult data from 2018 to 2021 indicated stable LSD use alongside significant increases in non-LSD hallucinogens, particularly substances with expanding medical applications (Livne et al., 2022). Comprehensive analysis of the most recent national data examining specific hallucinogen trajectories and their demographic correlates remains lacking. This study addresses this gap by examining hallucinogen use patterns from 2021 to 2023 using nationally representative NSDUH data, with particular focus on divergent trajectories between traditional psychedelics and dissociative agents across demographic subgroups commonly prioritized for prevention and access-focused interventions.

## 2 Methods

### 2.1 Data source and sample

We analyzed pooled 2021–2023 NSDUH data, providing nationally representative estimates among U.S. civilian population aged ≥ 12 years. The survey employs multistage area probability sampling with adolescent and young adult oversampling. Data collection utilized computer-assisted interviewing with ACASI for sensitive questions. Response rates ranged 44.1 %-46.9 %. The final sample included 173,808 respondents with complete data.

### 2.2 Measures

Past-year hallucinogen use was assessed through self-reported binary indicators for six substance categories: (1) LSD; (2) Ecstasy/Molly (MDMA); (3) phencyclidine (PCP); (4) ketamine; (5) Salvia divinorum; and (6) tryptamine psychedelics, including N,N-dimethyltryptamine (DMT), alpha-methyltryptamine (AMT), and 5-methoxy-N,N-diisopropyltryptamine (5-MeO-DiPT; ‘Foxy’). Overall hallucinogen use was defined as any past-year use of the above substances. For each substance, respondents who reported lifetime use were asked about use within the past 12 months.

Demographic covariates were selected based on established literature on substance use determinants and included: age categories (12–17, 18–25, 26–34, 35 + years); biological sex (male/female); race/ethnicity (non-Hispanic White, non-Hispanic Black, Hispanic, non-Hispanic Asian, other/multiracial); educational attainment (less than high school, high school graduate, some college, college graduate); annual family income (<$20,000, $20,000–49,999, $50,000–74,999, ≥$75,000); self-rated health status (excellent/very good, good, fair/poor); and health insurance coverage (private, public, uninsured).

### 2.3 Statistical analysis

All analyses incorporated NSDUH’s complex survey design. Multivariable logistic regression with survey year as continuous predictor estimated annual change odds. Models adjusted for demographics to estimate annual change in odds from 2021 to 2023. We examined four subgroups considered vulnerable: individuals in poverty, males, young adults (18–25), and uninsured individuals. These subgroup analyses were selected a priori because national epidemiologic studies consistently show higher hallucinogen use among males and young adults, and because poverty and lack of health insurance are markers of structural vulnerability linked to reduced access to health care and substance use treatment (Shalit et al., 2019, Keyes and Patrick, 2023, Olfson et al., 2022). Subgroup trend estimates were obtained from stratified models.

Secondary analyses examined demographic correlates of use for each substance, employing separate logistic regression models with all covariates entered simultaneously. To assess potential multicollinearity, we calculated variance inflation factors (VIF) for all predictors; all VIF values were < 5, indicating acceptable independence among covariates. Missing data on covariates (<0.3 % for any variable) were handled through listwise deletion. Sensitivity analyses using multiple imputation yielded virtually identical results.

All statistical tests were two-sided with significance set at p < 0.05. Analyses were conducted using Stata version 18.0 (StataCorp, College Station, TX), utilizing survey-specific procedures (svy commands) to account for the complex sampling design.

## 3 Results

### 3.1 Sample characteristics and overall prevalence

The weighted sample represented approximately 280 million U.S. individuals aged ≥ 12 years annually. Overall past-year hallucinogen use was stable: 2.83 % (95 % CI: 2.52–3.14) in 2021, 2.70 % (2.41–2.98) in 2022, and 2.82 % (2.52–3.12) in 2023 (Table 1).

**Table 1:** Past-Year Hallucinogen Use Prevalence (%, 95 % CI), NSDUH 2021–2023.

Year	LSD, % (95 % CI)	PCP, % (95 % CI)	Ecstasy/MDMA, % (95 % CI)	Ketamine, % (95 % CI)	Salvia, % (95 % CI)	DAF (tryptamines), % (95 % CI)	Any hallucinogen, % (95 % CI)
2021	0.88 (0.72–1.04)	0.06 (0.01–0.11)	0.82 (0.65–0.98)	1.61 (1.42–1.80)	0.02 (0.01–0.04)	0.22 (0.14–0.30)	2.83 (2.52–3.14)
2022	0.80 (0.68–0.91)	0.07 (0.01–0.13)	0.71 (0.58–0.85)	1.68 (1.48–1.88)	0.03 (0.01–0.05)	0.25 (0.15–0.36)	2.70 (2.41–2.98)
2023	0.58 (0.47–0.68)	0.04 (0.01–0.08)	0.77 (0.62–0.93)	1.91 (1.67–2.16)	0.03 (0.01–0.05)	0.22 (0.14–0.30)	2.82 (2.52–3.12)

Notes: Table 1 values are weighted past-year prevalence percentages with 95 % confidence intervals (CIs). Hallucinogen categories are not mutually exclusive. Table 2 reports odds ratios (ORs) and 95 % CIs from logistic regression models estimating the association between survey year (2021–2023; treated as continuous) and past-year use overall and within pre-specified subgroups. NSDUH =  National Survey on Drug Use and Health.

### 3.2 Temporal trends in hallucinogen use

Multivariable logistic regression revealed modest but not statistically significant increases in overall hallucinogen use from 2021 to 2023 (aOR=1.01, 95 % CI: 0.93–1.10, p = 0.84). However, this aggregate trend masked striking divergence across specific substances (Table 2).

**Table 2:** Associations Between Year (2021–2023) and Past-Year Use, Overall and by Subgroup: Adjusted Odds Ratios (aOR) and 95 % Confidence Intervals.

	**All Hallucinogen** aOR (95 % CI)		**LSD** aOR (95 % CI)	**PCP** aOR (95 % CI)
All sample (weighted)	1.009 p = 0.833 (0.928–1.097)		0.832*** p = 0.002 (0.745–0.929)	0.895 p = 0.691 (0.511–1.565)
Family income < $30,000	0.989 p = 0.830 (0.888–1.100)		0.809** p = 0.013 (0.685–0.955)	1.014 p = 0.970 (0.484–2.126)
Male	1.036 p = 0.429 (0.947–1.134)		0.760*** p = 0.000 (0.670–0.863)	0.764 p = 0.398 (0.405–1.440)
Age 18–25	0.861*** p = 0.006 (0.775–0.957)		0.705*** p = 0.000 (0.621–0.800)	0.547** p = 0.040 (0.308–0.973)
Without insurance	0.977 p = 0.811 (0.806–1.185)		0.802 p = 0.204 (0.568–1.132)	0.311* p = 0.092 (0.079–1.221)
	**Ecstasy** aOR (95 % CI)	**Ketamine** aOR (95 % CI)	**Salvia** aOR (95 % CI)	**Tryptamines** aOR (95 % CI)
All sample (weighted)	0.979 p = 0.789 (0.832–1.151)	1.106** p = 0.038 (1.006–1.216)	1.113 p = 0.626 (0.717–1.730)	1.034 p = 0.787 (0.809–1.321)
Family income < $30,000	0.955 p = 0.552 (0.820–1.113)	1.086 p = 0.186 (0.960–1.229)	1.427 p = 0.193 (0.830–2.453)	0.987 p = 0.937 (0.703–1.385)
Male	0.986 p = 0.881 (0.819–1.187)	1.159** p = 0.022 (1.023–1.314)	1.235 p = 0.462 (0.697–2.190)	0.994 p = 0.968 (0.720–1.372)
Age 18–25	0.897 p = 0.203 (0.758–1.062)	1.101 p = 0.204 (0.948–1.279)	1.078 p = 0.717 (0.713–1.630)	0.718 p = 0.105 (0.480–1.074)
Without insurance	0.911 p = 0.594 (0.643–1.291)	1.024 p = 0.831 (0.821–1.277)	0.954 p = 0.885 (0.500–1.820)	1.377 p = 0.219 (0.822–2.306)

Significance: *** p < 0.01; ** p < 0.05; * p < 0.10. aOR =  adjusted odds ratio.

LSD use declined significantly over time (aOR=0.83, 95 % CI: 0.75–0.93), decreasing from 0.88 % (0.72–1.04) in 2021–0.58 % (0.47–0.68) in 2023 (Table 1). In subgroup trend models, the decline was statistically significant among males, young adults, and uninsured respondents (Table 2).

Conversely, ketamine use increased significantly (aOR=1.11, 95 % CI: 1.02–1.21), rising from 1.61 % (1.42–1.80) in 2021–1.91 % (1.67–2.16) in 2023 (Table 1). In subgroup trend models, the increase was statistically significant among individuals below the federal poverty level and among uninsured respondents (Table 2).

Other hallucinogens showed variable patterns. Ecstasy use remained relatively stable (0.82 % [95 % CI: 0.70–0.94] in 2021 and 0.77 % [0.65–0.89] in 2023; Table 1), with no significant overall trend (aOR=0.98, 95 % CI: 0.83–1.15). PCP and Salvia use remained uncommon, and tryptamine psychedelics showed no significant overall change (aOR=1.03, 95 % CI: 0.81–1.32) (Table 1, Table 2).

### 3.3 Demographic correlates of use

Demographic patterns varied substantially across substances, revealing distinct individual profiles. Females demonstrated consistently lower odds of use across all substances, with adjusted odds ratios ranging from 0.33 (95 % CI: 0.12–0.86) for PCP to 0.64 (95 % CI: 0.53–0.77) for Ecstasy. Age patterns differed markedly: LSD and Salvia use peaked among 18–25 year-olds (aOR=1.03 and 8.06 versus 12–17 years, respectively), while ketamine use peaked among 26–34-year-olds (aOR=13.66, 95 % CI: 8.53–21.90).

Educational attainment showed the strongest associations, particularly for Ecstasy. College graduates had higher odds of Ecstasy use compared to those with less than high school education (aOR=1.89, 95 % CI: 1.21–2.93, p = 0.01). This educational gradient was evident but less pronounced for LSD (aOR=1.77, 95 % CI: 1.17–2.65, p = 0.01) and ketamine (aOR=1.77, 95 % CI: 1.33–2.36, p < 0.001).

Race/ethnicity patterns varied by substance. Non-Hispanic Whites showed highest odds of LSD use, while Ecstasy use was elevated among Hispanic and multiracial individuals. Ketamine use showed less pronounced racial/ethnic variation. Higher income was generally associated with lower odds of use after adjusting for education, suggesting complex socioeconomic patterns. Uninsured individuals had higher odds of ketamine use (aOR=1.50, 95 % CI: 1.18–1.91, p < 0.001) but not LSD use (aOR=1.27, 95 % CI: 0.96–1.68, p = 0.1).

## 4 Discussion

This analysis of nationally representative NSDUH data indicates stability in overall past-year hallucinogen use from 2021 to 2023, alongside substance-specific divergence: LSD use declined, whereas ketamine use increased (Table 1, Table 2). These opposing trajectories likely reflect differences in regulatory status, medical accessibility, and evolving perceptions of risk, including the rapid expansion of medically supervised ketamine treatment and broader diffusion of shorter-duration dissociative experiences (Weleff et al., 2024).

The 17 % annual decline in LSD use marks a reversal from earlier increases documented in prior NSDUH analyses. Past-year LSD use decreased from 0.88 % (95 % CI: 0.72–1.04) in 2021–0.58 % (0.47–0.68) in 2023 (Table 1), suggesting that the earlier period of increasing LSD uptake has ended and that individuals interested in hallucinogenic experiences may increasingly be choosing other substances such as ketamine or psilocybin (Johnson et al., 2018).

The concurrent rise in ketamine use—from 1.61 % (95 % CI: 1.42–1.80) in 2021–1.91 % (1.67–2.16) in 2023 (Table 1)—is consistent with increased visibility of ketamine in both clinical and nonmedical contexts. As ketamine is prescribed for depression and other indications, expanded availability may increase opportunities for diversion and nonmedical use, underscoring the need for clear clinical guidance, patient education, and harm-reduction messaging (Wilkinson et al., 2017).

Ketamine’s unique position as the only legally available hallucinogen for mental health treatment appears to be reshaping use patterns. Unlike classic psychedelics that remain Schedule I substances, ketamine’s medical legitimacy may reduce stigma and perceived risk. The shorter duration of ketamine experiences (1–2 h versus 8–12 h for LSD) and dissociative rather than psychedelic phenomenology may appeal to individuals seeking altered states while maintaining daily responsibilities (Dore et al., 2019).

The persistent educational gradient in hallucinogen use, particularly the approximately 1.8-fold higher odds of LSD use among college graduates, suggests that use remains concentrated among socioeconomically advantaged populations despite overall declining trends. This pattern may reflect differential access to harm reduction information, integration resources, and safe use settings. However, increasing ketamine use among economically disadvantaged groups indicates that dissociative agents may be following a different diffusion pattern, potentially driven by self-medication for untreated mental health conditions. The demographic correlates observed in our study are largely consistent with prior epidemiological research (Shalit et al., 2019, Keyes and Patrick, 2023, Davis et al., 2022, Yang et al., 2023); however, given the shifting landscape of use, ongoing research to identify emerging high-risk groups remains essential.

### 4.1 Clinical and policy implications

Healthcare providers should recognize increasing ketamine use, particularly among uninsured and economically disadvantaged patients who may present with complications. Unlike classic psychedelics, ketamine carries substantial misuse potential with tolerance, withdrawal, and compulsive use patterns (Schoevers et al., 2016). The concentration of ketamine increases among vulnerable populations warrants targeted interventions, including expanding insurance coverage for mental health services, developing clinical guidelines for ketamine prescribing and monitoring, and implementing surveillance systems to track adverse outcomes. Harm-reduction programs should incorporate ketamine-specific education addressing tolerance development, bladder toxicity, and dependence risk. Ensuring equitable access to evidence-based treatment while preventing misuse requires coordinated clinical and public health responses.

Divergent trends suggest that uniform regulatory approaches to hallucinogens may be insufficient. Current federal scheduling classifies most hallucinogens as Schedule I substances with no accepted medical use, yet ketamine (Schedule III) is now widely prescribed for depression, while psilocybin and MDMA are advancing through FDA approval pathways. Meanwhile, LSD use has declined despite similar therapeutic potential to psilocybin, highlighting how regulatory status and medical accessibility—rather than pharmacological properties alone—shape population-level use patterns. Policy frameworks should be updated to reflect the relative risks and therapeutic potential of specific substances rather than treating hallucinogens as a uniform class (Nutt, King and Phillips, 2010).

### 4.2 Limitations

Several limitations warrant consideration. First, NSDUH relies on self-reported data, which may underestimate use of stigmatized substances. Second, the cross-sectional design prevents analysis of individual trajectories over time. Third, the survey excludes homeless and incarcerated populations, who may have different use patterns. Fourth, NSDUH does not assess frequency, dosage, or context of use, limiting our understanding of consumption patterns and associated risks. Future research should employ longitudinal designs, examine the impact of state-level policy changes, and investigate health outcomes associated with specific hallucinogens. Qualitative research exploring motivations for substance choice—particularly factors driving potential shifts from LSD to ketamine—could inform targeted interventions (Morgan, 2012).

## 5 Conclusions

The U.S. hallucinogen landscape is experiencing shifting trends among specific substances, with use of traditional psychedelics like LSD declining while use of dissociative agents like ketamine is increasing, particularly among vulnerable populations. These divergent trajectories reflect the complex interplay of regulatory frameworks, medical legitimization, and evolving cultural attitudes toward consciousness-altering substances. As psychedelic medicine advances toward mainstream acceptance and ketamine treatment expands, comprehensive surveillance, evidence-based regulation, and equitable access to both treatment and harm reduction services will be essential for optimizing public health outcomes while minimizing potential harms. The increased use of ketamine among economically disadvantaged populations highlights the need for targeted interventions addressing both underlying mental health needs and substance-specific risks.

## Author disclosures

None

## CRediT authorship contribution statement

**Y. Tony Yang:** Writing – review & editing, Supervision, Project administration, Conceptualization. **Jing-Jer Chen:** Writing – original draft, Investigation, Data curation. **Berg Carla:** Writing – review & editing, Supervision.

## Ethics

This study analyzed publicly available, de-identified data and did not constitute human subjects research under our institutional policy; institutional review board review was not required.

## Declaration of Generative AI and AI-assisted technologies in the writing process

During manuscript preparation, the authors used an AI-assisted tool (e.g., ChatGPT) **only** for language refinement. After using the tool, the authors reviewed and edited the content as needed and take full responsibility for all content. *(If no AI tools were used, omit this paragraph.)*

## Role of the funder/sponsor

No sponsor or funder had any role in the study design; data collection, analysis, or interpretation; writing of the report; or the decision to submit the article for publication.

## Funding

This research did not receive any specific grant from funding agencies in the public, commercial, or not-for-profit sectors.

## Declaration of Competing Interest

The authors declare that they have no known competing financial interests or personal relationships that could have appeared to influence the work reported in this paper.

## Data Availability

The analysis uses de-identified, publicly available NSDUH microdata (2021–2023). Data and access information are provided by the study sponsor’s repository; no individual identifiers are included. (A brief data statement is requested at submission.)
